# Validity of diagnoses and procedures in Japanese dental claims data

**DOI:** 10.1186/s12913-021-07135-3

**Published:** 2021-10-18

**Authors:** Sachiko Ono, Miho Ishimaru, Yusuke Ida, Hayato Yamana, Yosuke Ono, Kazuto Hoshi, Hideo Yasunaga

**Affiliations:** 1 Department of Eat-loss Medicine, The University of Tokyo, 7-3-1 Hongo, Bunkyo-ku, Tokyo, 113-8655 Japan; 2 Department of Health Service Research, Faculty of Medicine, University of Tsukuba, 1-1-1 Tennodai, Tsukuba, Ibaraki, 305-8575 Japan; 3 Healthcare Executive Program, The University of Tokyo, 4F Administration Bldg., UTokyo Hospital, 7-3-1, Hongo, Bunkyo-ku, Tokyo, 113-0033 Japan; 4 Department of Health Service Research, The University of Tokyo, 7-3-1 Hongo, Bunkyo-ku, Tokyo, 113-0033 Japan; 5 Department of General Medicine, National Defense Medical College, 3-2 Namiki, Tokorozawa, Saitama, 359-8513 Japan; 6 Department of Sensory and Motor System Medicine, The University of Tokyo, The University of Tokyo Hospital Hongo 7-3-1, Bunkyo-ku, Tokyo, 113-0033 Japan; 7 Department of Clinical Epidemiology and Health Economics, School of Public Health, The University of Tokyo, 7-3-1 Hongo, Bunkyo-ku, Tokyo, 113-0033 Japan

**Keywords:** Dental claims data, General anesthesia, Oral cancer, Patient care, Teeth

## Abstract

**Background:**

Dental claims data have been used for epidemiological studies without establishing the validity of the recorded diagnoses or procedures. The present study aimed to examine the accuracy of diagnoses, procedures, operation time, and the number of teeth recorded in dental claims data.

**Methods:**

We reviewed the charts of 200 patients who visited and 100 patients who were hospitalized in the Department of General Dentistry, Orthodontics, and Oral and Maxillofacial Surgery in an academic hospital between August 2012 and December 2017. The sensitivity and specificity of the dental claims data for five diseases and 15 procedures were evaluated. We assessed the difference in the number of teeth and duration of general anesthesia between claims data and chart reviews.

**Results:**

Sensitivity was more than 86% for six out of seven diagnoses except for pericoronitis (67%). Specificity ranged from 72% (periodontal disease) to 100% (oral cancer for inpatient). The sensitivity of procedures ranged from 10% (scaling for inpatient) to 100%, and the specificity ranged from 6% (food intake on the day of the surgery) to 100%. The mean (standard deviation [SD]) number of teeth in the chart review was 22.6 (6.8), and in the dental claims was 21.6 (8.6). The mean (SD) operation time was 171.2 (120.3) minutes, while the duration of general anesthesia was 270.9 (171.3) minutes.

**Conclusions:**

The present study is the first study to validate dental claims data, and indicates the extent of usefulness of each diagnosis and procedure for future dental research using administrative data.

**Supplementary Information:**

The online version contains supplementary material available at 10.1186/s12913-021-07135-3.

## Introduction

In the era of big data, claims data are being increasingly utilized for medical and dental research [1–4]. The large sample size and variety of clinical data provided by claims data enables large-scale studies when a randomized controlled trial is infeasible. However, the credibility of these data; i.e., whether the data capture the patients’ actual health status, has been a major concern since claims data are recorded for reimbursement, not for research purposes. The importance of a validation study has been highlighted to avoid misclassification bias in study results [5].

In previous studies that examined the validity of Japanese claims data, the recorded diagnoses generally had high specificity and low sensitivity, while both procedures and pharmacy records tended to show high specificity and high sensitivity [6, 7]. However, to date, no validation study has been conducted for dental claims data despite the widespread application of such databases.

The aim of the present study was to examine the accuracy of diagnoses, procedures, operation time, and the number of teeth recorded in dental claims data in a single academic hospital. We conducted this validation study using chart and radiograph review results as reference standards.

## Method

### Data source

Various types of claims data are made by healthcare providers in the Japanese health insurance system, including medical, dental, and pharmacy claims data. Dental claims data include information on general dental practice and on oral and maxillofacial surgeries performed by dentists or dental hygienists.

We conducted the present study on patients who received dental treatment or oral and maxillofacial surgeries under the dental claims system in a Japanese academic hospital with 1264 beds. This hospital has a Department of General Dentistry, Orthodontics, and Oral and Maxillofacial Surgery, in which operations are performed on patients with oral cancer, benign tumors of the oral region, jaw deformities, jaw fractures, and cleft lip and palate. Dental procedures such as tooth extraction, dental checkup, and tooth cleaning for patients admitted to other departments of the hospital are also performed in this department. The claims and charts are available in an electronic format. Diseases and procedures are recorded using original Japanese codes, which are provided by the Ministry of Health, Labour, and Welfare for reimbursement purposes and have a finer granularity than the International Classification of Disease (ICD) codes.

### Study population and variables

We included patients admitted or who visited the Department of General Dentistry, Orthodontics, and Oral and Maxillofacial Surgery between August 2012 and December 2017. We excluded all data after 2018 for technical reasons. The electronic health record system in the university hospital where we conducted the study was renewed in the following year, and so we could not extract any data after 2018 in the same format as before. We randomly selected 200 outpatients and 100 hospitalized patients. In addition to these randomly selected patients, we identified patients with the following diagnoses in their dental claims using the Japanese diagnosis codes for reimbursement (Supplementary Table 1): medication related osteonecrosis of the jaw (MRONJ), pneumonia, surgical site infection, dysphagia, postoperative bleeding, and death. When the number of eligible patients in each diagnosis exceeded 100, we randomly selected 100 patients.

The items evaluated in the study are shown in Table 1. We evaluated five diagnoses, 15 procedures, the number of teeth, and the time of general anesthesia. The number of teeth was documented based on the diagnosis of periodontal disease using claims data. For patients with periodontal disease, we counted the number of teeth from 1 to 28, excluding the wisdom teeth. For hospitalized patients who underwent oral and maxillofacial surgery, we extracted the time of general anesthesia from the claims data. Although operation time was a focus of this study, it was recorded only in the medical chart and not in the claims record. Therefore, we compared the operation time from the medical records and the duration of anesthesia from the claims data to quantify the difference because researchers usually utilize the duration of anesthesia recorded in the claims data as a surrogate of operation time. For outpatients, we evaluated the items for a year from the time of their first visit; and for hospitalized patients, we evaluated the items from the time of their first admission to discharge. Additionally, we evaluated patients with the above-mentioned diagnoses (MRONJ, pneumonia, surgical site infection, dysphagia, and postoperative bleeding) and whose death had been recorded in the claims data by reviewing the chart.

**Table 1:** Items evaluated in the study

	Randomly selected outpatients (*n* = 200)	Randomly selected inpatients (*n* = 100)	Patients identified by the claims data for PPV calculation^a^
**Diagnoses**			
Oral cancer	◯	◯	
Jaw fracture	◯	◯	
Periodontal disease	◯		
Pericoronitis	◯		
The number of teeth	◯		
Temporomandibular disorders	◯		
Death			◯
Surgical site infection			◯
Dysphagia			◯
Medication related osteonecrosis of the jaw			◯
Pneumonia			◯
Bleeding			◯
**Procedures**			
Intubation		◯	
Food intake (postoperative)		◯	
Tube feeding		◯	
Time of general anesthesia/operation^b^		◯	
Tooth extraction	◯	◯	
Orthopantomography	◯		
Computed tomography	◯		
Rehabilitation for eating		◯	
Videoendoscopic evaluation of swallowing		◯	
Swallowing videofluorography		◯	
Caries treatment	◯		
Scaling	◯	◯	
Repairing of denture	◯		
Tissue conditioning	◯		
Oral splints	◯		
Sedation	◯		

*PPV* Positive predictive value

^a^Number of patients vary by item. ^b^Time of general anesthesia was obtained from the claims data, and time of operation was obtained from the chart review

### Chart reviews

We conducted chart reviews in the hospital from October 2019 to December 2020. Two authors (SO and MI) independently conducted chart reviews of the cases and identified whether the patients had received each diagnosis or underwent each procedure. When in dispute, they consulted a third review author (YI). The number of teeth was counted by orthopantomography, and ranged from 1 to 28, excluding wisdom teeth or impacted teeth. The operation time was obtained from the surgery record. The inter-reviewer agreements for the diagnoses and procedures before the discussion on the chart reviews were evaluated using kappa coefficients and categorized as follows: near-perfect (0.81–1.00), substantial (0.61–0.80), moderate (0.41–0.60), fair (0.21–0.40), or poor (0.00–0.20).

### Statistical analysis

The frequencies of the diagnoses and procedures were counted from both the chart reviews and claims data. The sensitivity and specificity of the dental claims data were calculated, with the chart review results as the reference standard. Sensitivity was defined as the proportion of patients who were identified as having/undergoing a disease/procedure in the claims data among those who were confirmed as having/undergoing that disease/procedure by the chart reviews. Specificity was defined as the proportion of patients who were identified as not having/not undergoing a disease/procedure in the claims data among those who were confirmed to not having/not having undergone that disease/procedure by the chart review.

We calculated the positive predictive value (PPV) of the following diagnoses: MRONJ, pneumonia, surgical site infection, dysphagia, postoperative bleeding, and death. PPV was defined as the proportion of patients who were assessed as having a disease by the chart review among those who were identified as having that disease in the claims data. We also calculated 95% confidence intervals (CIs) for all estimates of sensitivity, specificity, and PPV.

Furthermore, we presented the Bland-Altman plot [7], and calculated intraclass correlation coefficients (ICC) for the number of teeth, operation time, and duration of anesthesia. All statistical analyses were performed using R version 3.6.1 (R Foundation for Statistical Computing, Vienna, Austria).

### Standard protocol approvals, registrations, and patient consent

This study was performed in accordance with the Declaration of Helsinki. The study protocol was approved by the Ethics Review Board of the University of Tokyo (2019308NI), which deemed that written informed consent from the participants was unnecessary. An announcement about the study and the possibility of participants opting out of the survey was made through the hospital website.

## Results

### Overview and patient characteristics

**Fig. 1:**
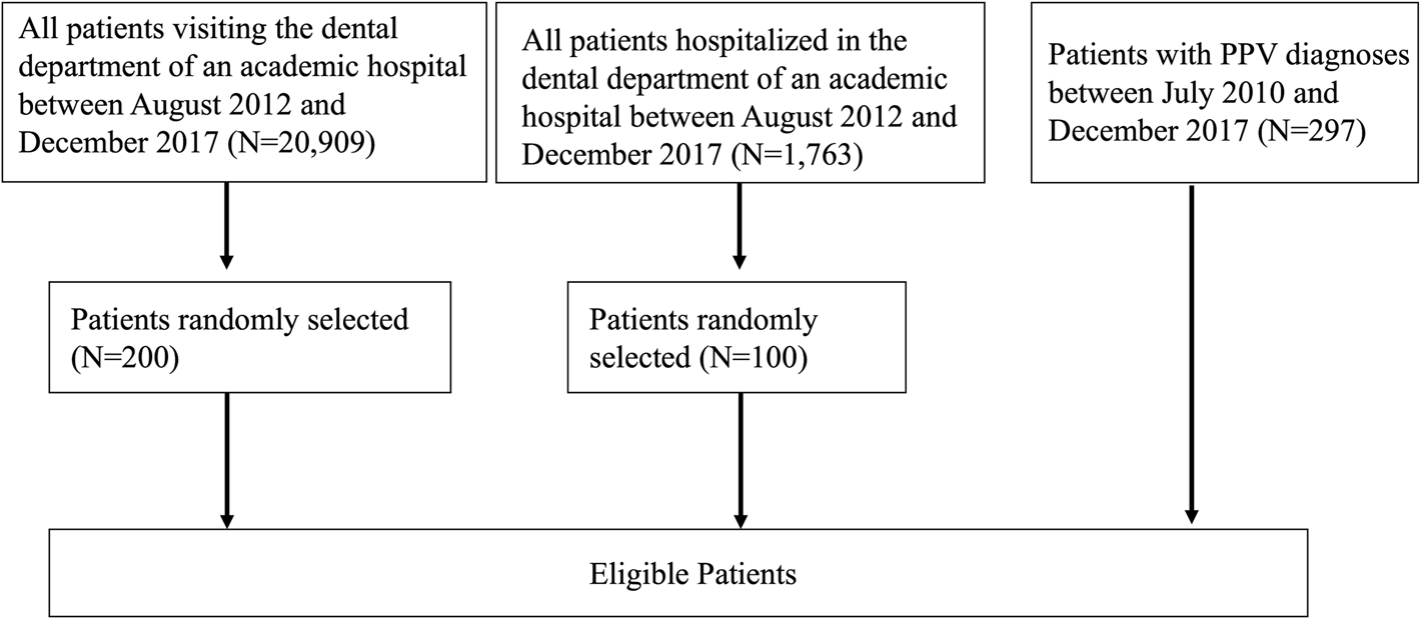
Flow chart of eligible patients. PPV, positive predictive value

We randomly extracted data for 200 outpatients and 100 hospitalized patients treated by oral and maxillofacial surgeons or dentists during the study period (Fig. 1). The numbers of patients whose data were extracted for the calculation of the PPV were 12 for death, 51 for surgical site infection, 34 for dysphagia, 14 for MRONJ, 87 for pneumonia, and 99 for bleeding.

The patients’ characteristics are shown in Table 2. The median age of the patients was 46.0 (interquartile range [IQR], 23.0–65.0) years for outpatients, and 29 (IQR, 15.8–33.5) years for hospitalized patients. The number of males among the outpatients and hospitalized patients was 103 (51.5%) and 44 (44.0%), respectively. Of the 100 hospitalized patients, 6, 14, 6, 13, 29, and 21 underwent surgery for cancer, benign tumor, fracture, sagittal split ramus osteotomy, cleft lip and palate, and other procedures, respectively.

**Table 2:** Characteristics of patients

Variables	Outpatients (*n* = 200)^a^	Inpatients (*n* = 100)^a^
Age, Median (IQR)	46.0 (23.0–65.0)	29.0 (15.8–33.5)
Male	103 (51.5)	44 (44.0)
Surgery		
Cancer	–	6 (6.0)
Benign tumor	–	14 (14.0)
Fracture	–	6 (6.0)
SSRO	–	13 (13.0)
CLP	–	29 (29.0)
Other	–	21 (21.0)

*CLP* Cleft lip and palate, *IQR* Interquartile range, *SSRO* Sagittal split ramus osteotomy

^a^Data are presented as n (%) except as otherwise indicated

### Validity of the diagnoses

The frequencies, inter-reviewer agreement, and validity indicators for the diagnoses are presented in Table 3. All inter-reviewer agreements for diagnoses of outpatients or hospitalized patients in the chart review were near-perfect even before discussions by the reviewers. In the chart review for determining the PPV of the diagnoses, the inter-reviewer agreement was near-perfect for one diagnosis, and substantial, moderate, and fair for two diagnoses each.

**Table 3:** Frequencies of diagnoses and validity indices for the dental claims-based diagnosis identification

	Eligible patients	Frequency (charts)		Inter-reviewer agreement (kappa)	Frequency (dental claim)		Sensitivity (95% CI)	Specificity (95% CI)	PPV (95% CI)	NPV (95% CI)
	n	n	(%)		n	(%)				
**Diagnoses**										
Oral cancer (outpatient)	200	5	(2.5)	1.00	10	(5.0)	1.00 (0.48, 1.00)	0.97 (0.94, 0.99)	0.50 (0.19, 0.81)	1.00 (0.98, 1.00)
Oral cancer (inpatient)	100	6	(6.0)	0.82	5	(5.0)	0.83 (0.36, 1.00)	1.00 (0.96, 1.00)	1.00 (0.48, 1.00)	0.99 (0.94, 1.00)
Jaw fracture (outpatient)	200	2	(1.0)	1.00	4	(2.0)	1.00 (0.16, 1.00)	0.99 (0.96, 1.00)	0.50 (0.07, 0.93)	1.00 (0.98, 1.00)
Jaw fracture (inpatient)	100	7	(7.0)	0.92	7	(7.0)	0.86 (0.42, 1.00)	0.99 (0.94, 1.00)	0.86 (0.42, 1.00)	0.99 (0.94, 1.00)
Periodontal disease	200	69	(34.5)	0.91	98	(49.0)	0.88 (0.78, 0.95)	0.72 (0.63, 0.79)	0.62 (0.52, 0.72)	0.92 (0.85, 0.97)
Pericoronitis	200	24	(12.0)	0.93	24	(12.0)	0.67 (0.45, 0.84)	0.95 (0.91, 0.98)	0.67 (0.45, 0.84)	0.95 (0.91, 0.98)
Temporomandibular disorders	200	7	(3.5)	1.00	26	(13.0)	0.86 (0.42, 1.00)	0.90 (0.84, 0.94)	0.23 (0.09, 0.44)	0.99 (0.97, 1.00)
**Diagnoses (PPV)**										
Death	12	2		0.63	12				0.16 (−0.04, 0.37)	
Surgical site infection	51	16		0.22	51				0.31 (0.19, 0.44)	
Dysphagia	34	27		0.59	34				0.79 (0.66, 0.93)	
MRONJ	14	13		1.00	14				0.93 (0.79, 1.06)	
Pneumonia	87	12		0.44	87				0.14 (0.06, 0.21)	
Bleeding	99	76		0.70	99				0.77 (0.69, 0.85)	

*MRONJ* Medication related osteonecrosis of the jaw, *NPV* Negative predictive value, *PPV* Positive predictive value

Sensitivity was more than 86% for six out of seven diagnoses (oral cancer for inpatient and outpatient, jaw fracture for inpatient and outpatient, periodontal disease, and temporomandibular disorder), except for pericoronitis (67%). Specificity ranged from 72% (periodontal disease) to 100% (oral cancer for inpatient). The PPV ranged from 13.8% (pneumonia) to 100% (oral cancer for inpatient).

### Validity of procedures

The frequencies, inter-reviewer agreement, and validity of the procedures are presented in Table 4. No patient underwent rehabilitation for eating, swallowing videofluorography, or sedation, and only one patient underwent videoendoscopic evaluation of swallowing. Except for these four procedures, the inter-reviewer agreement for procedures before discussion by the reviewers was near-perfect for 12 procedures, substantial for two procedures, moderate for one procedure, and poor for one procedure.

**Table 4:** Frequencies of procedures and validity indices for the dental claims-based procedure identification

	Eligible patients	Frequency (charts)		Inter-reviewer agreement (kappa)	Frequency (dental claim)		Sensitivity (95% CI)	Specificity (95% CI)	PPV (95% CI)	NPV (95% CI)
	n	n	(%)		n	(%)				
**Procedures**										
Intubation	100	11	(11.0)	0.66	11	(11.0)	0.82 (0.48, 0.98)	0.98 (0.92, 1.00)	0.82 (0.48, 0.98)	0.98 (0.92, 1.00)
Food intake (postoperative)										
Day 0	80	9	(11.3)	0.84	75	(93.8)	0.89 (0.52, 1.00)	0.06 (0.02, 0.14)	0.11 (0.05, 0.20)	0.80 (0.28, 0.99)
Day 1	80	75	(93.8)	0.97	75	(93.8)	0.95 (0.87,0.99)	0.20 (0.01,0.72)	0.95 (0.87, 0.99)	0.20 (0.01, 0.72)
Day 2	78	73	(93.6)	0.97	73	(97.3)	0.95 (0.87, 0.98)	0.20 (0.01, 0.72)	0.95 (0.87, 0.99)	0.20 (0.01, 0.72)
Day 3	73	67	(91.8)	1.00	68	(93.2)	0.94 (0.85, 0.98)	0.17 (0.00, 0.64)	0.93 (0.84, 0.98)	0.20 (0.01, 0.72)
Tube feeding	100	20	(20.0)	1.00	24	(24.0)	0.90 (0.68, 0.99)	0.93 (0.84, 0.97)	0.75 (0.53, 0.90)	0.97 (0.91, 1.00)
Tooth extraction (outpatient)	200	47	(24.5)	0.96	49	(24.5)	1.00 (0.92, 1.00)	0.99 (0.95, 1.00)	0.96 (0.86, 1.00)	1.00 (0.98, 1.00)
Tooth extraction (inpatient)	100	18	(18.0)	0.79	12	(12.0)	0.61 (0.36, 0.83)	0.99 (0.93, 1.00)	0.92 (0.62, 1.00)	0.92 (0.84, 0.97)
Orthopantomography	200	113	(56.5)	0.99	127	(63.5)	0.99 (0.95, 1.00)	0.83 (0.73, 0.90)	0.88 (0.81, 0.93)	0.99 (0.93, 1.00)
Computed tomography	200	15	(7.5)	1.00	15	(7.5)	1.00 (0.79, 1.00)	1.00 (0.98, 1.00)	1.00 (0.79, 1.00)	1.00 (0.98, 1.00)
Rehabilitation for eating	100	0	(0.0)	–	0	(0.0)	–	–	–	–
Videoendoscopic evaluation of swallowing	100	1	(1.0)	1.00	0	(0.0)	0	0	0	0
Swallowing videofluorography	100	0	(0.0)	–	0	(0.0)	–	–	–	–
Caries treatment	200	6	(3.0)	0.92	7	(3.5)	0.83 (0.36, 1.00)	0.99 (0.96, 1.00)	0.71 (0.29, 0.96)	0.99 (0.97, 1.00)
Scaling (outpatient)	200	44	(44.0)	0.93	32	(16.0)	0.73 (0.57, 0.85)	1.00 (0.98, 1.00)	1.00 (0.89, 1.00)	0.93 (0.88, 0.96)
Scaling (inpatient)	100	42	(42.0)	0.91	6	(6.0)	0.10 (0.03, 0.23)	0.97 (0.88, 1.00)	0.67 (0.22, 0.96)	0.60 (0.49, 0.70)
Repairing of denture	200	4	(2.0)	0.49	5	(2.5)	1.00 (0.40, 1.00)	0.99 (0.97, 1.00)	0.80 (0.28, 0.99)	1.00 (0.98, 1.00)
Tissue conditioning	200	3	(1.5)	1.00	3	(1.5)	0.67 (0.09, 0.99)	0.99 (0.97, 1.00)	0.67 (0.09, 0.99)	0.99 (0.97, 1.00)
Oral splints	200	2	(1.0)	0.00	3	(1.5)	1.00 (0.16, 1.00)	0.99 (0.97, 1.00)	0.67 (0.09, 0.99)	1.00 (0.98, 1.00)
Sedation	200	0	(0.0)	–	0	(0.0)	–	–	–	–

*NPV* Negative predictive value, *PPV* Positive predictive value

Sensitivity ranged from 10% (scaling for inpatient) to 100% (tooth extraction for outpatient, computed tomography, repairing of denture, and oral splint). Procedures with sensitivity below 80% were tooth extraction for inpatient (61%), scaling for outpatient (73%), scaling for inpatient (10%), and tissue conditioning (67%). Similarly, specificity ranged from 6% (food intake on the day of the surgery) to 100% (computed tomography and scaling for outpatients). The only procedure with a specificity below 80% was postoperative food intake (6–20%). The PPV ranged from 10% (food intake on the day of the surgery) to 100% (computed tomography and scaling for outpatients). The procedures with a PPV less than 80% were food intake on the day of the surgery (11%), caries treatment (71%), scaling for inpatient (67%), tissue conditioning (67%), and oral splint (67%).

### Validity of the number of teeth and the time of general anesthesia

**Fig. 2:**
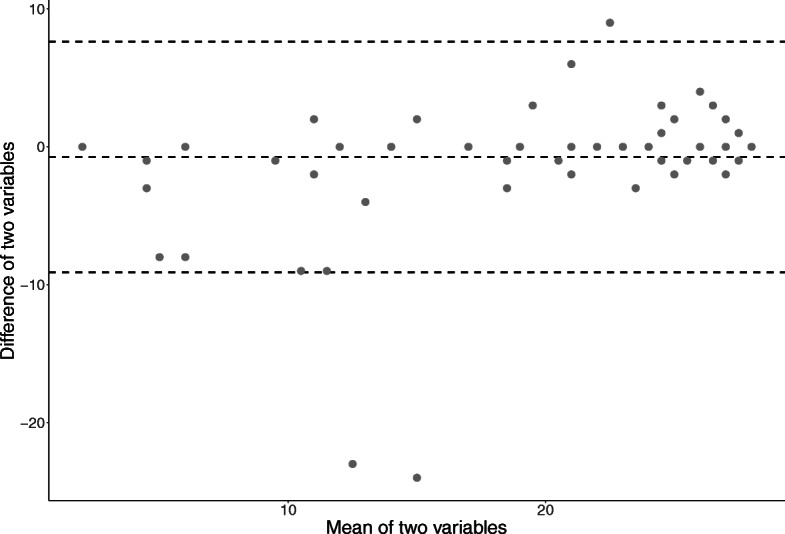
Discrepancy in the number of teeth between dental claims data and chart review

The number of teeth, operation time in the charts, and duration of general anesthesia recorded in the dental claims are shown in Table 5. The ICC between reviewers was 0.995 for the number of teeth and 0.996 for the duration of anesthesia. The mean (standard deviation [SD]) number of teeth in the chart review was 22.6 (6.8), while the mean (SD) number of teeth in dental claims was 21.6 (8.6). The difference in the number of teeth between the chart review and the dental claim was 1.0, and the ICC was 0.841. The Bland-Altman plot for the number of teeth is shown in Fig. 2. The discrepancy in the number of teeth between the chart and dental claims was consistent regardless of the actual number of teeth.

**Table 5:** Validity of number of teeth and duration of surgery for the dental claims-based identification

	Eligible patients	Charts		ICC (inter-reviewer)	Dental claim		Difference	ICC (chart-claim)
	n	Mean	(SD)		Mean	(SD)		
The number of teeth	148	22.6	(6.8)	0.995	21.6	(8.6)	1.0	0.841
Duration of operation/general anesthesia	80	171.2	(120.3)	0.996	270.9	(171.3)	99.7	0.599

*ICC* Intraclass correlation coefficient, *SD* Standard deviation

**Fig. 3:**
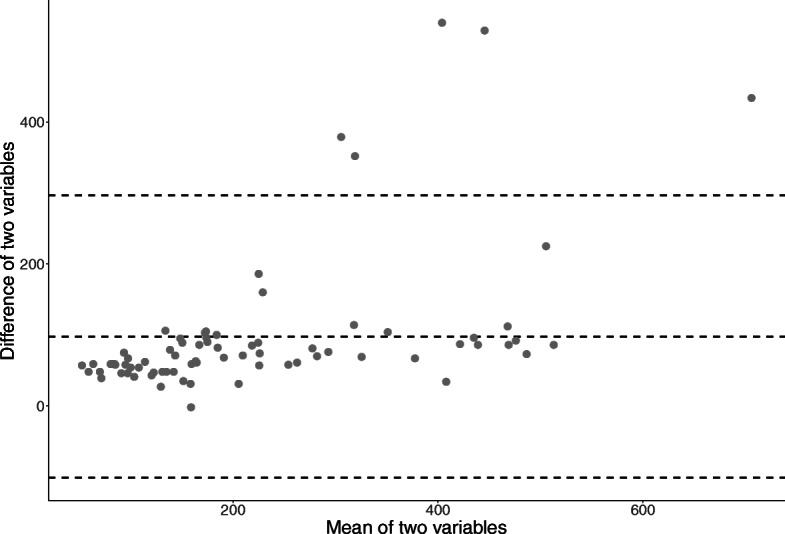
Bland-Altman plot showing discrepancy in the operation time and general anesthesia

The mean (SD) operation time was 171.2 (120.3) minutes, while the mean (SD) duration of general anesthesia was 270.9 (171.3) minutes. The difference in duration between operation and general anesthesia was 99.7 min, and the ICC was 0.599. The Bland-Altman plot for the duration of operation/general anesthesia is shown in Fig. 3. Although the operation time was always shorter than the duration of anesthesia, the discrepancy increased as the duration of anesthesia was prolonged.

## Discussion

In the present study, we evaluated the validity of dental diagnoses, dental procedures, number of teeth, and duration of anesthesia recorded in claims data, using chart review as the reference standard.

### Validity of diagnoses

The sensitivity and specificity of the diagnoses were generally high, with the exception of the sensitivity for the diagnosis of pericoronitis and specificity for the diagnosis of periodontal disease. Regarding the low sensitivity for the diagnosis of pericoronitis, we looked for signs of actual inflammation of the tissue surrounding a third molar when identifying the disease from the charts. However, pericoronitis was recorded in the dental claim when a patient had a history of pericoronitis with no current symptoms and was often unrecorded when no subsequent third molar extraction had been planned or conducted. Regarding the low specificity for the diagnosis of periodontal disease, the diagnoses may have been registered in the dental claims simply because the current health care system in Japan requires specific diagnoses, including periodontal disease, to be recorded to perform an orthopantomography. The PPVs of the target diagnoses, including death, surgical site infection, and pneumonia, were lower than those of MRONJ, dysphagia, and bleeding. According to a previous systematic review, the PPV of bisphosphonate-related osteonecrosis of the jaw, which is a part of MRONJ, was 74.5% using the ICD-10 code of K10.2 [8]. The PPV of MRONJ in our study was 92.9%, which was higher than the PPV obtained in the systematic review. Since MRONJ in our study was defined with the fine granularity accorded by the original Japanese disease codes for MRONJ and bisphosphonate-osteonecrosis of the jaw, the PPV of MRONJ in our study may be more accurate. Compared to two previous studies that assessed Japanese medical claims [9, 10] and found a PPV of 95.6–95.7% for death, the PPV value of death in our study was very low. Most cases with incorrect death records were probably attributable to human error while recording data. Except for deaths caused by oral cancer, deaths may not be usually recorded in dental claims data. Our results suggest that researchers should be cautious when death has been listed as an outcome in dental claims data.

Our results suggest that the diagnoses of systemic diseases may be less accurate than those of oral/dental diseases in dental claims, although the exact mechanism of this is unclear. Since exacerbated systemic conditions are likely to be treated at other departments specializing in the disease, extracting medical claims from these departments may help identify these systemic conditions more accurately [9, 10].

### Validity of procedures

Sensitivity and specificity of the procedures were high, with the exception of sensitivity for the procedure of scaling for inpatients and specificity for the procedure of food intake on the day of the surgery. Although dental scaling is common for patients undergoing oral surgery, the procedure may be unclaimed because the reimbursement for the procedure requires other time-consuming tests such as pocket depth examination, along with considerable paperwork. The low specificity for the procedure of food intake on the day of the surgery can be explained by the uncertainty of the patient’s condition after oral surgery: the meal ordered in advance for the day of the surgery in case the patient was well enough to eat after a minor surgery may have remained on record despite non-consumption. Another possibility is that the day of surgery was flexible/undetermined, and thus, the meal could not be canceled beforehand. In either case, researchers should be aware of this discrepancy when using the duration of fasting as an outcome in dental research.

### Validity of the number of teeth and time of general anesthesia

The number of teeth recorded in dental claims was comparable to the actual number of teeth found in the orthopantomography. However, there were a few cases with 20 or more discrepancies between the dental claims data and the actual number of teeth, as illustrated in the Bland-Altman plot. The discrepancy in the number of teeth probably arose from the method used to count teeth in dental claims. We counted the number of teeth recorded by the tooth numbering system attached to the diagnosis of periodontal disease, which is the most common initial diagnosis for patients undergoing first examination. However, in the case of acute periodontitis, the dentist may have entered only a part of the dentition with the symptoms and diagnosis.

We compared the operation time and duration of anesthesia because researchers usually utilize duration of anesthesia recorded in the claims data as a surrogate of operation time. The difference between the operation time and duration of anesthesia seemed stable with a slight positive deviation for a longer operation time. The consistent time difference between the two variables was probably a result of the introduction of and awakening from general anesthesia, as oral and maxillofacial surgeries are generally performed without additional anesthetic procedures such as epidurals or spinal anesthesia. The slightly greater difference in longer operation times can be explained by delayed awakening from anesthesia for prolonged surgery [11] or ICU transfer after a complicated surgery.

### Strengths and limitations of our study

This study is the first to examine the accuracy of diagnoses, procedures, operation time, and the number of teeth recorded in dental claims data in Japan. A strength of our research is the random selection of outpatients and inpatients. By adopting random sampling, we were able to conduct a more accurate validation study of diagnoses and procedures. The present study may be useful for researchers using dental claims data in their studies. To date, researchers have conducted epidemiological studies without determining the validity of dental claims data [1, 12, 13]. As a result, these studies may have had a misclassification bias in their included diagnoses or procedures. We observed high sensitivity and specificity for almost all diagnoses, procedures, and the number of teeth in the dental claims data, barring a few exceptions. Thus, our study can be seen as a fundamental study that assures the quality of previous and future research utilizing dental claims data in Japan.

However, this study also had several limitations. First, the study was conducted in a single academic hospital. Although the annual number of dental patients is larger than that in community dental clinics, the results may be difficult to extrapolate to other institutions. Second, incomplete information in the chart was an issue encountered in the study. We used unformatted chart review as the reference standard because there was no other available source fulfilling the purpose of this study. Inter-rater agreement presented as kappa statistics between two reviewers was low in some variables due to differences in interpretation of the free text in the chart. Although kappa statistics are affected by prevalence and tend to present small values in cases of low prevalence [14], we still acknowledge the difficulty of identifying patients’ true conditions based on the chart review due to unrecorded clinical information. Third, our sample size may be insufficient for an accurate validation of some rarely performed dental procedures such as rehabilitation for eating, videoendoscopic evaluation of swallowing, swallowing videofluorography, and sedation. Hence, future studies involving multiple institutions and a larger sample size are necessary.

## Conclusion

This is the first study to investigate the validity of dental diseases and procedures in dental claims data with chart review as the reference standard. The results indicated the extent of usefulness of each diagnosis and procedure for future dental research using administrative data. Because this was a single-center study, future studies involving multiple institutions are necessary.

## Supplementary Information

**Additional file 1.**

## Data Availability

The medical charts and claims generated and analyzed during the current study are not publicly available because of ethical and legal restrictions in Japan (Amended Act on the Protection of Personal Information, December 2016, ver. 2). The medical charts and claims belong to each patient and are managed by the University of Tokyo Hospital. To access the datasets, a researcher needs to be a member of the University of Tokyo Hospital, to have permission from the hospital, and to have ethical approval from The Ethics Review Board of the University of Tokyo. (Contact form of the University of Tokyo Hospital, https://www.ut-crescent.jp/patients/contact/).

## Abbreviations

CLP: Cleft lip and palate
ICC: Intraclass correlation coefficient
IQR: Interquartile range
NPV: Negative predictive value
MRONJ: Medication related osteonecrosis of the jaw
PPV: Positive predictive value
SD: Standard deviation
SSRO: Sagittal split ramus osteotomy
