# Supplementary material for: Validity of diagnoses and procedures in Japanese dental claims data
Source: BMC Health Serv Res. 2021 Oct 18;21:1116. doi: 10.1186/s12913-021-07135-3 (PMC8525021; doi:10.1186/s12913-021-07135-3)
Supplement: Supplementary file 1 — Additional file 1. [file 12913_2021_7135_MOESM1_ESM.doc]

Supplementally table 1. The definition of the disease codes for each diagnosis

| Periodontal disease | 5233004,5233007,5233014,5234009,5234013,5234016,5234028,5235002,  8839667,8839996,8842275,8843129,8843292,8843327,8843442,8843447,  8843614,8843615,8843616,8843617 |
| --- | --- |
| Pericoronitis | 5233009,8843663 |
| Oral cancer | 1409003,1419002,1420003,1421002,1422003,1429007,1439002,1449002,  1459007,1460003,1469004,1479001,1489003,1490001,1600003,1602001,  1609002,1619005,1732005,1950005,1950008,8831084,8831237,8831889,  8832258,8832563,8833363,8833368,8833400,8833430,8833609,8834279,  8835381,8835397,8835450,8835477,8835961,8836428,8836431,8836443,  8836461,8837362,8837552,8838349,8840249,8840960,8841093,8842556,  8842594,8842603,8843068,8843079,8843434,8843436,8843437,8843438,  8844128,8844153,8846359,8846403,8844125,8843320,1701003,1988009,  8833002,8833010 |
| Jaw fracture | 8022002,8831101,8831111,8831133,8835379,8841737,8844013,8844015,  8844017,8844018,8844019,8831845,8028009,8028010 |
| Temporomandibular disorders | 5249004,7169004,7964002,8831850,8831851,8831852,8842352,8843286,  8848128,8848163,8848165 |
| Surgical site infection | 6829010,8799006,8830118,8845681,9985006 |
| Dysphagia | 7872003,8844884,8846478 |
| Medication related osteonecrosis of the jaw | 8849512,8848247,8848303 |
| Pneumonia | 8838802,8830718,8838795,8841070,4824001,8830122,8847070,8830065,  8841197,8837370,8830932,8836058,8839998,8846063,8846406,4829003,  8832171,8839549,4819003,8837800,8832646,8841647,4830007,4860018,  4860030,4860043,4860045,8834801,8838435,50700038830602,8834867  8838214,8838416,8841219,8840982,8833161,8847570,5183002,5183011,  5183012,8830397,8830866,8847878,9973011 |
| Bleeding | 5258013,8833916 |

Supplementally table 2. The definition of the procedure codes for each procedure.

| Intubation | 309009110,309009310,140009310,140009550,140009750,140023510,  140023750,140023950,140030830,140031430,140039550,140039650 |
| --- | --- |
| Food intake (postoperative) | 320000110,320000110,320000270,320000370,320000410,320000410,  320000510,320000910,320001110,320001210,320001310,320001410,  320001510,320001710,320001810,320001910,320002010,320002110,  320002210,320002410,320002510,320002610,320003410,320003510,  320003610,320003710,320003810,320003910,320004010,320004210,  320004310,320004410,320004510,320004610,320004710,320004810,  320004910,320005010,320005110,320005210,320005310,320005410,  320005510,197000110,197000110,197000710,197000710,197000810,  197000910,197001010,197001110,197001210,197001210,197001310,  197001310,197001710,197002010,197002110,197002210,197002310, 197002710,197002810,197002910,197003110,197003210,197003310,  197003410,197003510,197003610,197005110,197005210 |
| Tube feeding | 140023210,140023350,309008810 |
| Time of general anesthesia | 150332510,150332610,150332710,150332810,150332910,150333010,  150333110,150333210,150233410,150328210 |
| Tooth extraction | 310000110,310000210,310000310,310000410,310000510,310000710,  150110010,150110110,150110210,150110310,150110410 |
| Orthopantomography | 305000410 |
| Computed tomography | 170011710,170011710,170011810,170028610,170033410,170033410,  170034910,305004910,305005050,305005110 |
| Rehabilitation for eating | 308000510,308000510,308004310,180016610,180016610,180054310 |
| Videoendoscopic evaluation of swallowing | 170028510,170028510 |
| Swallowing videofluorography | 160187510 |
| Caries treatment | 309000110,309001710,313001210,313001310,313002010,313002210,  313009350,313009450,313009550,313009650,313009720,313009820,  313009920,313010020,313010120,313010220,313010320,313014050,  313023550,313024310,313024410,313024510,313024610,313025150,  313025250,313025450,313025550,313026020,313026020,313026120,  313026120,313026220,313026220,313026320,313026320,313031320,  313031420,313031520,313031620 |
| Scaling | 309004810,309005010,309005110,309005210 |
| Repairing of denture | 313021610,313021770 |
| Tissue conditioning | 309008310 |
| Oral splints | 309006710,309006810,309006910,309007050,309007050,309007150,  309007150,309010350,309010350,309010450,309010450,309014910,  309018650,309018750 |
| Sedation | 311000310,311000510,150232110,150232210,150232210,150232210,  150332410,150332410,150370710 |

| Intubation | 309009110,309009310,140009310,140009550,140009750,140023510,  140023750,140023950,140030830,140031430,140039550,140039650 |
| --- | --- |
| Food intake (postoperative) | 320000110,320000110,320000270,320000370,320000410,320000410,  320000510,320000910,320001110,320001210,320001310,320001410,  320001510,320001710,320001810,320001910,320002010,320002110,  320002210,320002410,320002510,320002610,320003410,320003510,  320003610,320003710,320003810,320003910,320004010,320004210,  320004310,320004410,320004510,320004610,320004710,320004810,  320004910,320005010,320005110,320005210,320005310,320005410,  320005510,197000110,197000110,197000710,197000710,197000810,  197000910,197001010,197001110,197001210,197001210,197001310,  197001310,197001710,197002010,197002110,197002210,197002310, 197002710,197002810,197002910,197003110,197003210,197003310,  197003410,197003510,197003610,197005110,197005210 |
| Tube feeding | 140023210,140023350,309008810 |
| Time of general anesthesia | 150332510,150332610,150332710,150332810,150332910,150333010,  150333110,150333210,150233410,150328210 |
| Tooth extraction | 310000110,310000210,310000310,310000410,310000510,310000710,  150110010,150110110,150110210,150110310,150110410 |
| Orthopantomography |  |
| Computed tomography | 170011710,170011710,170011810,170028610,170033410,170033410,  170034910,305004910,305005050,305005110 |
| Rehabilitation for eating | 308000510,308000510,308004310,180016610,180016610,180054310 |
| Videoendoscopic evaluation of swallowing | 170028510,170028510 |
| Swallowing videofluorography | 160187510 |
| Caries treatment | 309000110,309001710,313001210,313001310,313002010,313002210,  313009350,313009450,313009550,313009650,313009720,313009820,  313009920,313010020,313010120,313010220,313010320,313014050,  313023550,313024310,313024410,313024510,313024610,313025150,  313025250,313025450,313025550,313026020,313026020,313026120,  313026120,313026220,313026220,313026320,313026320,313031320,  313031420,313031520,313031620 |
| Scaling | 309004810,309005010,309005110,309005210 |
| Repairing of denture | 313021610,313021770 |
| Oral splints | 309006710,309006810,309006910,309007050,309007050,309007150,  309007150,309010350,309010350,309010450,309010450,309014910,  309018650,309018750 |
| Sedation | 311000310,311000510,150232110,150232210,150232210,150232210,  150332410,150332410,150370710 |
